# Stages and costs of processing donated corneas for transplantation in eye banks: a scoping review

**DOI:** 10.1007/s10561-026-10245-z

**Published:** 2026-08-03

**Authors:** Bethânia Karoline Alvaro Menezes, Marcos Antonio Ferreira Junior, Aline Moraes da Silva, Giovanna Encinas Fernandes, Felipe Machado Mota, Oleci Pereira Frota, Matilde Delmina da Silva Martins

**Affiliations:** 1 Federal University of Mato Grosso Do Sul (UFMS), Integrated Health Institute, Instituto Integrado de Saúde (INISA), Cidade Universitária, Av. Costa E Silva, S/N Pioneiros, Campo Grande, Mato Grosso Do Sul 79070-900 Brazil; 2 Bragança Polytechnic University (IPB), Bragança, Portugal

**Keywords:** Eye banks, Costs, Scoping review, Corneal transplantation

## Abstract

The objective was to map the stages and costs of processing donated corneal tissue for transplantation in Eye Banks. The method followed the JBI Institute Reviewer’s Manual, and the PRISMA-ScR guidelines were adopted for reporting, to answer the question: “What are the stages and costs of processing donated corneal tissue for transplantation in Eye Banks?” Studies considered for inclusion comprised qualitative, quantitative, mixed-method, observational, experimental, quasi-experimental studies, reviews, institutional documents, manuals, theses, and dissertations, all available in full text and open access, with no restrictions on language or publication period. Information sources included PubMed, Embase, Cochrane Library, Scopus, Web of Science, ScienceDirect, CINAHL (EBSCOhost), the Virtual Health Library, and gray literature databases. The systematic search resulted in the final inclusion of seven studies. The results identified an operational workflow comprising three main phases: corneal selection and recovery, processing and evaluation, and the pre-surgical stage. The review revealed extensive variation in evaluation and discard/disqualification protocols across institutions and countries, especially in those with decentralized regulation. A lack of cost analyses and economic data was identified. In conclusion, although the processing workflow was mapped, the lack of protocol uniformity and, above all, the absence of cost data limit the understanding of financial impact and the sustainability of Eye Banks, reinforcing the need for future research with an economic focus and the development of standardized indicators.

**Supplementary Information:**

The online version contains supplementary material available at https://doi.org/10.1007/s10561-026-10245-z.

## Introduction

Corneal transplantation, or keratoplasty, consists of replacing pathologic or non-functional corneal tissue that has lost its optical properties, even when biologically viable, with healthy donor corneal tissue, when other clinical requirements for transplantation are met. It is one of the most frequently performed and successful allogeneic procedures worldwide (Bowling 2016). Corneal failure may result from hereditary or acquired causes. Examples of hereditary causes include genetic dystrophies and degenerations such as keratoconus, whereas acquired causes include infections, burns, ulcers, and trauma (Cruz et al. 2017; Garcia et al. 2017).

To ensure quality and safety, donated corneas undergo rigorous processing steps in Eye Banks (EB). Globally, EB play an essential role in donor recovery, tissue processing, and distribution of corneas for transplantation. However, regulatory models and quality standards vary among countries. In the United States, the Eye Bank Association of America (EBAA) (EBAA 2026) establishes national accreditation and auditing standards, ensuring tissue standardization and traceability (Lambert; Chamberlain 2017). In Canada, the system is similarly centralized, with oversight conducted by national health authorities (EBAA 2026).

In the European context, Directive 2004/23/EC defines minimum quality and safety criteria for human tissues and cells. In addition, professional organizations such as the European Eye Bank Association (EEBA) contribute to the advancement of eye banking practices through technical exchange, education, and professional collaboration, although implementation and monitoring still vary among Member States (EEBA 2026; European Parliament 2004). In other contexts, India and Brazil share similar regulatory characteristics, with national guidelines complemented by regional regulations, resulting in heterogeneity in practice and sanitary oversight (Christy et al. 2023).

With emphasis on the Brazilian context, the operation of these institutions is governed by Law No. 9.434/1997, which addresses the removal of organs, tissues, and body parts for transplantation, and by Collegiate Board Resolutions (RDC) No. 67/2008 and No. 55/2015 of the National Health Surveillance Agency (ANVISA), which define minimum technical and sanitary requirements to ensure the quality of tissues intended for therapeutic use (Brasil 1997; Brasil 2008; Brasil 2015).

Despite these national Brazilian guidelines, EB are also subject to state legislation and local norms, which result in regional variations in procedures and may compromise standardization within the national transplantation system (Brasil 2008).

EB are responsible for establishing protocols for corneal quality classification. They use clinical criteria evaluated through slit-lamp examination, applying scoring systems that determine whether a tissue is suitable for optical or therapeutic transplantation, or should be discarded/disqualified (ANVISA 2022; Brasil 2015; Cruz et al. 2022). With scientific advances, this evaluation can also be performed through other methods, such as specular microscopy and optical coherence tomography (OCT) (Chaurasia, Vanathi 2021; Chong et al. 2024; Han et al. 2024).

At all stages, hospital supplies and specialized equipment are required, generating operational costs that are poorly documented. However, scientific literature lacks studies that quantify or analyze these costs in EB, and this gap limits knowledge and hinders efficient management of these institutions (Almeida 2018).

Given this knowledge gap, there is a need for a comprehensive synthesis of evidence on the processing of donated corneas and the associated costs, to guide policy, establish technical standardization, and support financial planning. Therefore, the objective of this study was to map the stages and costs of processing donated corneal tissue for transplantation in EB.

## Methods

This scoping review was conducted according to the methodology proposed in the JBI Institute Reviewer’s Manual (Aromataris et al. 2024; Peters et al. 2020) and written in accordance with the Preferred Reporting Items for Systematic Reviews and Meta-Analyses extension for Scoping Reviews (PRISMA-ScR) guidelines (Tricco et al. 2018). The review protocol was previously registered on the Open Science Framework (OSF) and is available at https://doi.org/10.17605/OSF.IO/HGY8S.

To comprehensively and systematically map the available evidence on the stages and costs of processing donated corneal tissue for transplantation in EB, a scoping review design was chosen due to the exploratory nature of the topic and the expected heterogeneity among studies. The research process was structured into interdependent steps, including formulation of the research question, definition of eligibility criteria, development of the search strategy, selection of sources, data extraction, data analysis, and presentation of results.

### Search strategy

The research question was developed using the Population, Concept, and Context (PCC) mnemonic. The population included studies addressing keratoplasty or corneal transplantation. Regarding the concept, two central elements were used: processing and costs. The first refers to the stages that ensure the quality and safety of ocular tissue, from donor recovery to transplantation, in accordance with national and international regulations, including ANVISA resolutions (Brasil 2008; Brasil 2015) and the guidelines of international associations such as the Asian Eye Bank Association (AEBA) (AEBA 2026), the Eye Bank Association of America (EBAA) (EBAA 2026]), the European Eye Bank Association (EEBA) (EEBA 2026), and the European Directorate for the Quality of Medicines and HealthCare (EDQM) (EDQM 2019).

The second concept pertains to cost, understood as the amount of financial resources required to perform activities related to tissue processing. The context of interest encompasses exclusively EB, specialized institutions responsible for the collection, processing, and distribution of ocular tissues for transplantation. Thus, the following research question was formulated: “What are the stages and costs of processing donated corneal tissue for transplantation in Eye Banks?”.

### Eligibility criteria

Studies employing different methodological approaches were included: qualitative, quantitative, and mixed methods, as well as experimental, quasi-experimental, observational studies, and reviews, in addition to institutional documents, guides, manuals, theses, and dissertations.

No language restrictions or publication time limits were applied. Opinion articles, editorials, abstracts, conference proceedings, interviews, and studies addressing stages or costs unrelated to EB were excluded.

The preliminary search was conducted in PubMed, Embase, and the Cochrane Library to identify existing reviews and assess the feasibility of the study. Search strategies were developed using Medical Subject Headings (MeSH) and Emtree descriptors. The search syntaxes were: corneal transplantation OR keratoplasty AND eye bank AND procedure, and corneal transplantation OR keratoplasty AND eye bank AND methods. The searches were conducted in May 2025 and yielded 2.432 records in PubMed, 3.325 in Embase, and 1.474 in the Cochrane Library.

Subsequently, the strategy was expanded to Scopus, Web of Science, and the Virtual Health Library (VHL), in addition to sources of gray literature such as Google Scholar, the Networked Digital Library of Theses and Dissertations (NDLTD), BASE, and Open Access Theses and Dissertations, as well as government websites and regulatory documents on the topic.

### Study selection and data management

The search results were exported in the “RIS,” “BibTeX,” and “NBIB” formats. Duplicate removal was performed using Mendeley®, and the records were then imported into Rayyan QCRI® (Ouzzani et al. 2016), where blinded screening of titles and abstracts was conducted by two independent reviewers, supervised by a third. Discrepancies between reviewers were resolved by consensus.

The selection process followed three stages: initial screening of titles and abstracts; eligibility assessment with full-text reading of a sample for reviewer calibration; and final inclusion of studies after complete reading. It is important to note that, due to the exploratory and comprehensive nature of scoping reviews, methodological quality appraisal was not performed, in accordance with guidance indicating that the focus is on mapping the scope rather than assessing internal validity.

### Data extraction and synthesis

Data extraction was performed independently by two reviewers using a spreadsheet created in Microsoft Excel® for this purpose and adapted from the JBI manual. The following information was collected from the studies: language, year, origin, setting, objective, method, surgical techniques, main findings, identified stages, costs, limitations, and additional considerations.

## Results

The systematic search conducted in the Cochrane Library, CINAHL via the EBSCOhost platform, Embase, PubMed, ScienceDirect, Scopus, Web of Science, and the Virtual Health Library identified a total of 10.392 records. After removing 7.528 duplicates and 196 ineligible records identified by automated tools, 2.668 studies were screened by title and abstract. Of these, 2.618 were excluded for not meeting the predefined inclusion criteria, and 50 studies were included for full-text review.

After full-text reading of the 50 studies, six were excluded for meeting exclusion criteria. Among the 44 articles assessed for eligibility, 39 were excluded for not directly addressing the topic or for providing insufficient coverage. Thus, five studies were initially included in the review.

**Fig. 1:**
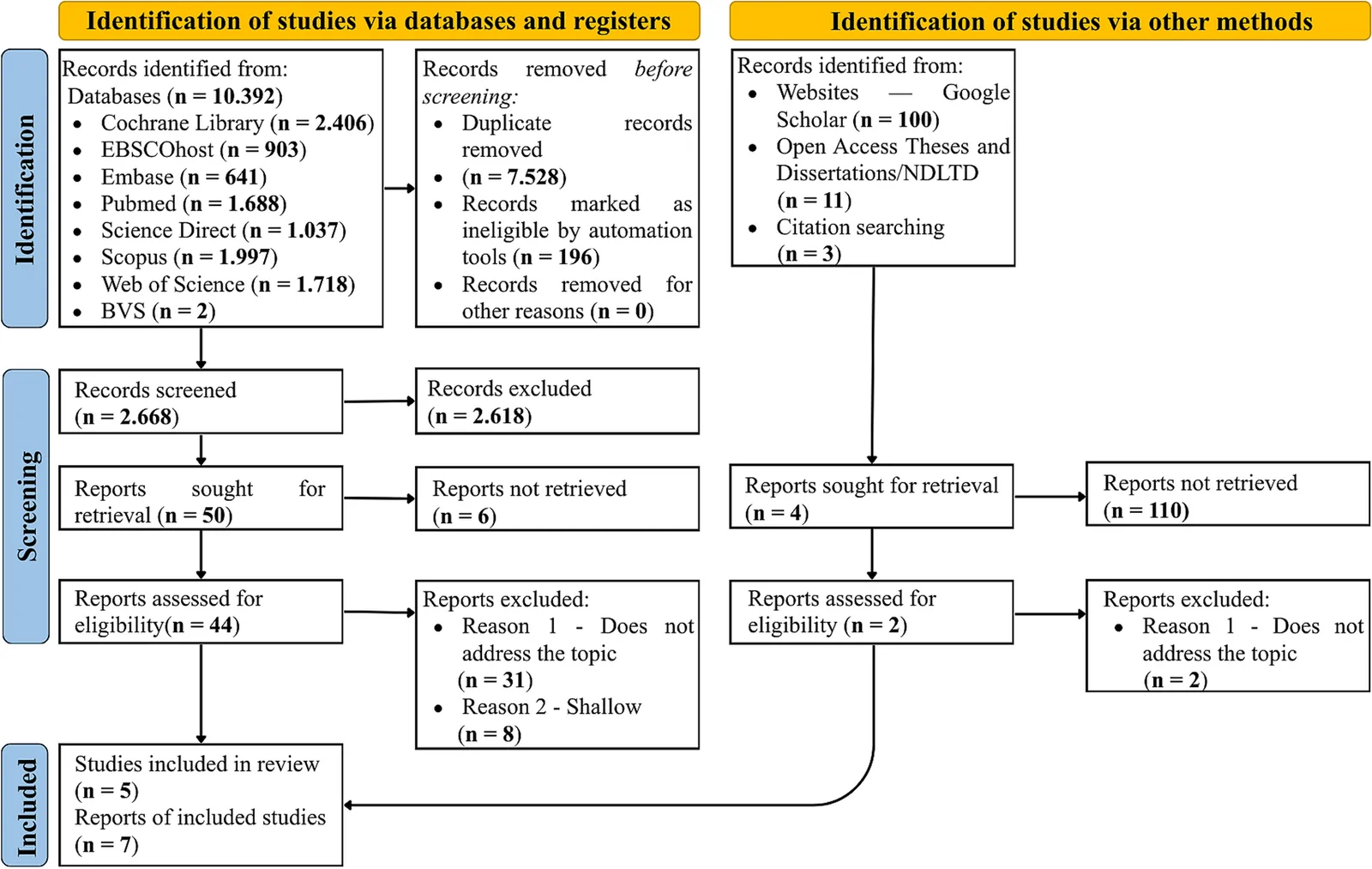
PRISMA Extension for Scoping Reviews (PRISMA-ScR) Flowchart Diagram (Tricco et al. 2018)

A manual search of the reference lists of selected articles and gray literature identified an additional 114 records, of which two were included after full-text reading, resulting in a final sample of seven studies. The process of identification, screening, eligibility assessment, and inclusion is illustrated in the PRISMA flowchart (Fig. 1).

The included studies were published between 1990 and 2025. Most were published in English (85,7%) and indexed in PubMed/Medline (71,4%). Regarding publication type, review articles predominated (57,1%).

The studies addressed different aspects of EB, with emphasis on the evaluation of donated tissue quality (Romano et al. 2024; Menezes et al. 2024; Anitha et al. 2023; Patil et al. 2022; Moshirfar et al. 2021; Freire et al. 2014), methods of preservation and storage (Moshirfar et al. 2021; Romano et al. 2024; Anitha et al. 2023; Menezes et al. 2024; Patil et al. 2022; Saini et al. 1996), donor selection criteria (Romano et al. 2024; Moshirfar et al. 2021; Menezes et al. 2024; Anitha et al. 2023; Freire et al. 2014), health regulation (Freire et al. 2014; Anitha et al. 2023; Romano et al. 2024; Moshirfar et al. 2021) and emerging technological trends (Anitha et al. 2023), such as the use of specular microscopy (Patil et al. 2022; Moshirfar et al. 2021; Anitha et al. 2023; Romano et al. 2024; Freire et al. 2014), automated endothelial cell assessment (Patil et al. 2022; Moshirfar et al. 2021; Romano et al. 2024; Freire et al. 2014) and bioengineering techniques applied to the cornea (Anitha et al. 2023; Moshirfar et al. 2021; Romano et al. 2024).

In several articles, concerns were noted regarding the standardization of processes (Romano et al. 2024; Anitha et al. 2023; Menezes et al. 2024) and the need for unified national and international protocols (Anitha et al. 2023; Romano et al. 2024). Other studies emphasized the importance of professional training (Menezes et al. 2024; Anitha et al. 2023) and continuing education (Anitha et al. 2023; Moshirfar et al. 2021; Romano et al. 2024) as key factors for ensuring quality and safety in ocular tissue processing (Menezes et al. 2024; Anitha et al. 2023; Moshirfar et al. 2021; Romano et al. 2024; Freire et al. 2014).

Table 1 presents the characterization of the studies included in this review.

**Table 1:** General study characteristics, Campo Grande/MS, Brazil, 2025. (n = 07)

Study/Year of publication	Country/Language	Type of Study	Objective	Stages in the EB	Costs in the EB
Anitha; et al. (2023)	India / English	Review article	Discuss the evolving role of EB	Recovery Tissue processing Distribution Donor screening Serological testing Evaluation (slit-lamp, specular microscopy) Pre-cut/preloaded preparation Quality Assurance (QA)	N/A
Freire; et al. (2014)	Brazil / English	Observational study	Describe the utilization of corneas recovered and processed for transplantation in an EB	Recovery Tissue processing Evaluation and selection (specular microscopy and slit-lamp) Serology	N/A
Menezes; et al. (2024)	Brazil / English	Narrative review	To present a narrative review of the most commonly described forms of EB organization and of the corneal transplantation process reported in the literature	Recovery Processing Evaluation Classification Storage Appropriate distribution of ocular tissues	N/A
Moshirfar; et al. (2021)	United States of America / English	Review article	To outline current practices and guidelines for corneal donation and EB. To describe the implications of COVID-19 and emerging diseases on the donor pool. To discuss future trends aimed at improving and increasing the efficiency of the processes involved	Screening Serological testing Physical assessment and donor history Tissue evaluation Preservation and storage Distribution	N/A
Patil; et al. (2022)	India / English	Prospective study	To qualitatively and quantitatively evaluate donor corneal tissue using slit-lamp and specular microscopy	Medical history collection (study focus) Preservation and storage Evaluation	N/A
Romano; et al. (2024)	Italy / English	Review article, descriptive synthesis	To describe the donor screening process for recovery teams, EB technicians, and ophthalmic surgeons	Tissue analysis and processing Preservation methods; microbiological evaluation Tissue quality assessment Preparation of lamellar grafts	N/A
Saini; et al. (1996)	India / English	Review article	To summarize issues related to restrictions on donor eye recovery, strategies to overcome them, EBs structures, and safe tissue recovery techniques	Preliminary procedures Blood collection Preparation Tissue recovery Preservation/storage Tissue evaluation	N/A

*N/A: Not Applicable. EB: Eye Banks. COVID-19: Coronavirus Disease 2019

Table 2 presents the stages identified in Table 1 in sequence and describes, according to the authors, the understanding of each step of the process. This organization provides a detailed view of how the different publications address the critical phases that ensure the quality and safety of corneal tissue.

**Table 2:** Organization and description of the steps of the process, Campo Grande/MS, Brazil, 2025. (n = 07)

Articles	Process stage	Description
Anitha; et al. (2023); Freire; et al. (2014); Menezes; et al. (2024); Saini; et al. (1996)	Recovery of Tissues	Initial procedures for obtaining and collecting ocular tissue from the deceased donor, including the planning and execution of the recovery process
Anitha; et al. (2023); Moshirfar; et al. (2021); Patil; et al. (2022)	Donor Screening/Selection	Collection of medical history, physical assessment, and determination of donor eligibility to prevent disease transmission or exclude low-quality tissues
Anitha; et al. (2023); Freire; et al. (2014); Moshirfar; et al. (2021); Romano; et al. (2024); Saini; et al. (1996)	Eligibility Testing (Serology and Microbiology)	Performance of mandatory serological tests (blood collection) and microbiological assessments to ensure tissue safety against infectious agents
Anitha; et al. (2023); Freire; et al. (2014); Menezes; et al. (2024); Romano; et al. (2024)	Tissue Processing	Manipulation of the tissue in the EB after recovery, which may include preparation of the corneoscleral disc under aseptic conditions
Menezes; et al. (2024); Moshirfar; et al. (2021); Patil; et al. (2022); Romano; et al. (2024); Saini; et al. (1996)	Preservation/Storage	Use of media and methods (such as Optisol, Cornisol, or organ culture) to maintain tissue viability and quality, particularly endothelial cell integrity, over a defined storage period
Anitha; et al. (2023); Freire; et al. (2014); Menezes; et al. (2024); Moshirfar; et al. (2021); Patil; et al. (2022); Romano; et al. (2024); Saini; et al. (1996)	Quality Assessment (General and Technical)	Detailed tissue inspection using specular microscopy and slit-lamp examination to assess endothelial cell density and morphology, as well as overall tissue integrity
Anitha; et al. (2023); Romano; et al. (2024)	Specialized Preparation (Pre-cut)	Advanced techniques for preparing lamellar grafts, such as pre-cut or preloaded corneas, aimed at facilitating the use of more modern surgical procedures (DMEK/DSAEK)
Anitha; et al. (2023); Menezes; et al. (2024)	Classification and Quality Assurance (QA)	Final classification of the tissue and implementation of QA programs for continuous monitoring of all processes
Anitha; et al. (2023); Menezes; et al. (2024); Moshirfar; et al. (2021)	Distribution	Organized and traceable delivery of viable tissue to corneal surgeons, often supported by an equitable allocation system

**Fig. 2:**
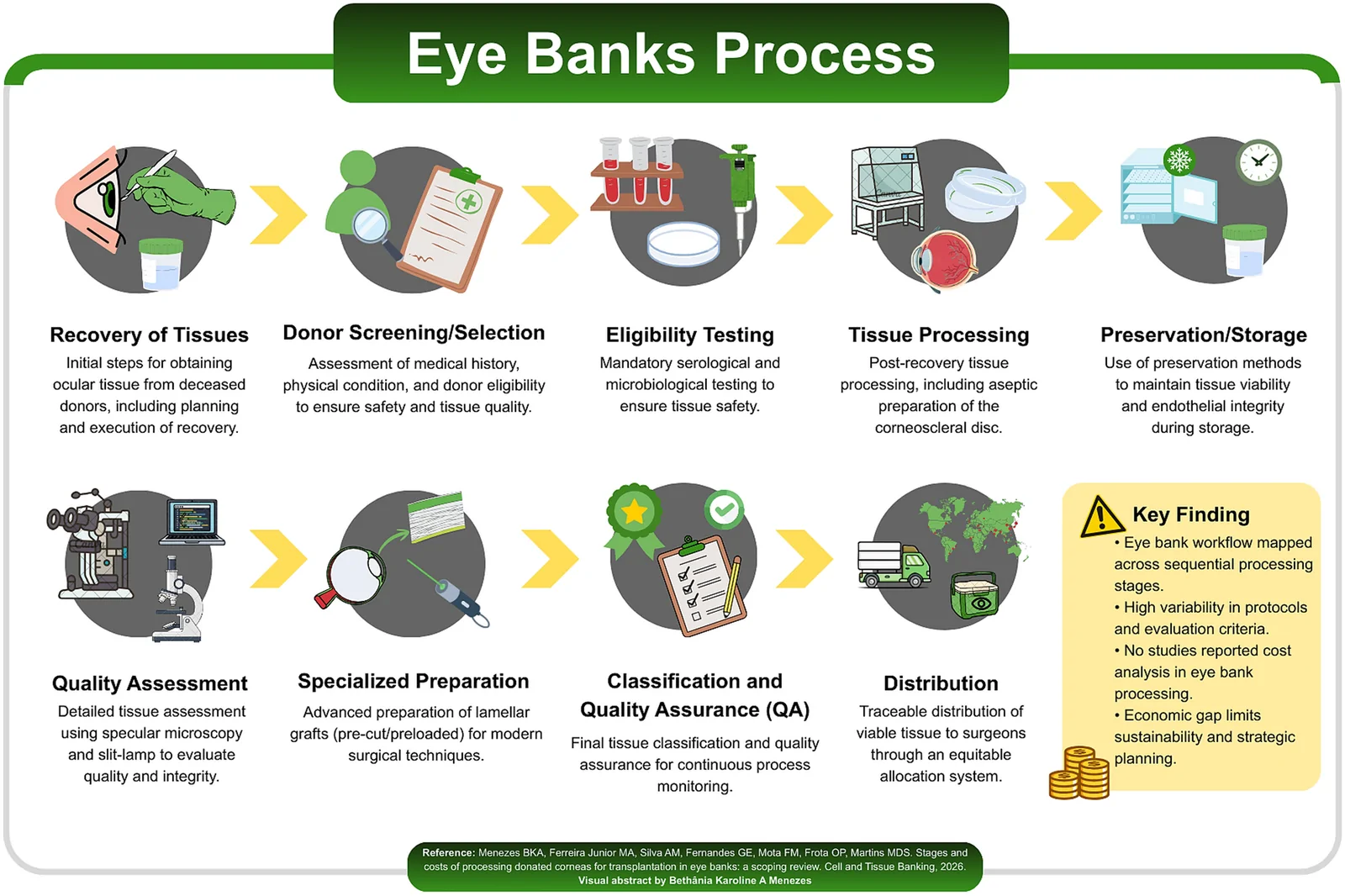
Workflow of corneal tissue processing in eye banks

The sequential stages of eye bank processing identified across studies are summarized in Fig. 2.

## Discussion

### Structure and stages of corneal processing

The studies included in this review made it possible to map the operational workflow of EB, organized into three main phases: corneal selection and recovery, processing with evaluation, and the pre-surgical stage. This structure, highlighted primarily by Romano et al. (2024), Moshirfar et al. (2021), and Menezes et al. (2024), reflects an international consensus on the essential stages required to maintain corneal quality for donation and to guide the tissue from donor identification to its release for transplantation.

The initial phase of donor selection and recovery involves clinical and serological screening of the donor, obtaining family consent, and tissue retrieval, generally through enucleation or corneoscleral excision. This stage is critical for ensuring the biological safety of the graft and was mentioned in 42,9% of the studies analyzed (Anitha et al. 2023; Freire et al. 2014; Saini et al. 1996). The literature indicates that delays in tissue recovery and inconsistencies in initial screening directly affect endothelial viability (Gram et al. 2022), underscoring the importance of standardized timelines and recovery protocols.

In the processing and evaluation phase, carried out within EB, laboratory procedures for morphological and microbiological analysis of the tissue were predominant. Assessment of endothelial cell density using specular microscopy and transparency evaluation through slit-lamp examination were common steps across studies. However, methodologies varied widely among institutions and countries, both in tissue quality classification and in discard criteria. This lack of uniformity represents a significant barrier to the comparability of results and to the rational use of available tissues (Lambert; Chamberlain 2017; Romano et al. 2024; Patil et al. 2022).

The pre-surgical stage, although less frequently addressed, is described as the final moment of quality verification before transplantation. In this stage, the surgeon is responsible for inspecting the storage medium and confirming corneal integrity to ensure that the tissue remains viable up to the moment of implantation (Moshirfar et al. 2021). This phase closes the tissue traceability cycle and reinforces the need for integration between EB and transplant teams (Ker et al. 2018).

### Standardization and quality in EB

The lack of standardization was a cross-cutting finding across all included studies. Although regulations issued by entities such as the Eye Bank Association of America (EBAA), the European Eye Bank Association (EEBA) and the European Directorate for the Quality of Medicines & HealthCare (EDQM) exist (EBAA 2026; EEBA 2026; EDQM 2019), their implementation varies substantially. In countries with decentralized regulation, such as Brazil and India (Brasil 2008; Brasil 2015; Christy et al. 2023), discrepancies in screening, preservation, and discard protocols are more evident, resulting in reduced tissue utilization and loss of operational efficiency (Romano et al. 2024; Menezes et al. 2024).

Freire et al. (2014) and Sousa and Sousa (2018) emphasize that process standardization is not limited to processing techniques but also involves the training of specialized teams and the maintenance of tissue traceability. The literature suggests that EB that adopt uniform protocols show lower discard and disqualification rates and greater predictability in the availability of suitable tissues. This reinforces the direct relationship between technical quality and administrative efficiency (Acharya et al. 2018; Laun et al. 2022; Wykrota et al. 2022).

Furthermore, guidelines such as the European Directive 2004/23/EC and EBAA standards detail the steps of screening, testing, processing, and traceability (European Parliament 2004; EBAA 2026), whereas in Brazil, ANVISA’s RDC No. 67/2008 and RDC No. 55/2015 establish minimum technical and sanitary requirements (Brasil 2008; Brasil 2015). Nonetheless, the persistent lack of practical standardization and comparable indicators compromises both the performance of EB and the development of evidence-based public policies (Acharya et al. 2018).

### Costs and sustainability of EB

The analysis of the studies reveals a critical scarcity of information regarding direct and indirect costs. All seven articles, despite addressing challenges related to funding and sustainability, failed to present quantifiable economic data. This lack of accounting transparency and cost metrics reflects a relevant methodological gap and limits a full understanding of the economic impact of processing practices.

Operational and supply costs represent notable barriers to the functioning of EB, especially in developing countries, where the cost of equipment and storage media is consistently cited as critical (Anitha et al. 2023). Regarding supplies, the Optisol-GS preservation medium was reported to be approximately ten times more expensive than the conventional McCarey-Kaufman (MK) medium (Patil et al. 2022).

Moreover, although the preparation of grafts by EB, whether pre-cut or pre-stripped, offers advantages such as reduced surgical time and the availability of tissues validated with documented quality control, these grafts result in a significantly higher acquisition cost per patient compared with those prepared intraoperatively (Pagano et al. 2021).

Among the factors influencing economic sustainability in these institutions, donor profile appears to be particularly relevant. Hatzfeld et al. (2024) reported that in their study on the medical and economic impact of corneas from elderly donors in France, costs ranged from €395 to €1,244. This variation has a direct impact on financial viability: donors aged 80 years or younger generated a mean net profit per cornea, whereas donors over 80 years resulted in a mean net loss for the EB. These findings highlight the complexity and variability of costs incurred in these services and reinforce the importance of cost analyses for setting priorities and guiding resource allocation decisions in planning (Bertram et al. 2016).

In a notable contrast, the tissue-processing model of the United States, recognized globally for its volume and efficiency in corneal processing, demonstrates a completely different operational reality. The high rate of corneal transplants per capita and the consolidation of the EB system reflect a large-scale, high-volume infrastructure (Gain et al. 2016). This scenario of high efficiency and high throughput, supported by substantial, standardized operational costs, does not translate to countries with more limited budgetary capacity. National and regional literature still lacks studies that structure cost analyses across each processing stage, hindering strategic planning and sustainability assessment for EB (Maidana et al. 2025).

In addition to direct economic challenges, the lack of uniformity in evaluation and discard criteria, particularly in countries with decentralized regulation, such as Brazil, has significant financial implications. Studies indicate that operational non-standardization compromises EB efficiency and increases the cost per effectively transplanted tissue due to higher loss rates and the need for reprocessing in laboratory stages (Romano et al. 2024; Menezes et al. 2024; Freire et al. 2014). This variability in protocols directly affects tissue utilization and the predictability of supply, which in turn impacts the overall cost per viable cornea (Acharya et al. 2018; Laun et al. 2022).

Furthermore, the literature highlights that EB with harmonized protocols show lower discard rates and greater administrative efficiency (Acharya et al. 2019; Wykrota et al. 2022; Sousa and Sousa 2018), reinforcing the direct relationship between technical quality and financial sustainability. Therefore, standardization should not be viewed solely as a technical requirement but as a strategic component essential for economic management and the long-term sustainability of EB operating under different regulatory contexts.

This study presents methodological limitations that must be considered when interpreting the results. Although a scoping review allows for a broad mapping of the literature, this approach does not include an in-depth assessment of the methodological quality of the studies, which may influence the robustness of the identified evidence. The heterogeneity of study designs, data collection methods, and operational definitions used by the included studies also limits direct comparison across findings.

Furthermore, the absence of international standardization for reporting stages and costs in EB hinders data synthesis and may have resulted in the omission of relevant information. Finally, the reliance on available documents and the potential omission of non-indexed studies or those published in institutional sources may have restricted the scope of the search and impacted the completeness of the mapping performed.

Nevertheless, a major strength of this review is its unprecedented and organized synthesis of corneal processing stages in EB, bringing together fragmented and heterogeneous evidence into a coherent and comparable structure. Even with the limited availability of economic analyses, the study advances the field by highlighting critical gaps related to the lack of standardization, the impact of these variations on tissue utilization, and the absence of unified indicators that integrate technical, operational, and financial dimensions. By systematically mapping these elements, the review expands the understanding of EB processes and guides priorities for future research.

## Conclusion

This scoping review made it possible to map the workflow of stages in EB, which includes corneal selection and recovery, processing with tissue evaluation, and pre-surgical steps. It was observed that although technological and regulatory advances exist, protocols still vary widely across institutions and countries, especially in contexts with decentralized regulation.

Such methodological differences compromise the comparability of results, tissue utilization, and the overall efficiency of services. A critical gap identified is the absence of systematized data on the costs associated with each stage of processing. None of the included studies presented economic analyses, which limits the understanding of the financial impact of current practices and hinders the adoption of planning and sustainability strategies within EB.

In addition, it is recommended that future studies integrate economic analyses into the technical evaluation of Eye Banks (EB), with a focus on the cost of technologies, operational scale, and preservation processes. The development of standardized indicators that integrate clinical, operational, and financial aspects may enhance comparability across institutions, strengthen the transplant network, and support more effective public policies that promote equity in resource allocation and encourage the harmonization of practices worldwide.

## Supplementary Information

Below is the link to the electronic supplementary material.

Supplementary file1 (DOCX 23 KB)

## Data Availability

No datasets were generated or analysed during the current study.
