# Supplementary material for: Stages and costs of processing donated corneas for transplantation in eye banks: a scoping review
Source: Cell Tissue Bank. 2026 Aug 3;27(3):42. doi: 10.1007/s10561-026-10245-z (PMC13433669; doi:10.1007/s10561-026-10245-z)
Supplement: Supplementary file 1 [file 10561_2026_10245_MOESM1_ESM.docx]

**METHOD OF LITERATURE SEARCH**

A comprehensive literature search was conducted in 2025 across multiple electronic databases, including PubMed, Embase, Scopus, Web of Science, Cochrane Library, EBSCOhost, ScienceDirect, BVS, and additional sources such as Google Scholar and Open Access Theses and Dissertations (NDLTD). The search strategies combined controlled vocabulary and free-text terms related to corneal transplantation, eye banks, processing methods, procedures, and costs. During the search process, different combinations of search terms were tested within each database to identify those yielding the most relevant and comprehensive results. The final combinations selected for this review, as well as the corresponding databases, search dates (day/month), and number of retrieved records, are presented in in the tables.

**Table 1.** Acronyms and controlled vocabulary (MESH), Campo Grande/MS, Brazil, 2025.

| ACRONYM | | MeSH |
| --- | --- | --- |
| P | Corneal transplant | - Corneal Transplantation - Keratoplasty |
| C | Processing (procedure or method) and cost | - Procedure - Methods - Processing |
|  |  | - Costs |
| C | Eye Banks | - Eye Banks |

**Table 2.** Search term combinations and number of records retrieved from electronic databases, Campo Grande/MS, Brazil, 2025.

| BASE | DATA | CROSSING | NUMBER OF ARTICLES |
| --- | --- | --- | --- |
| PUBMED | 11/06 – Search 2 | (((Corneal Transplantation) OR (Keratoplasty)) AND (Procedure)) AND (Eye Banks) | 717 |
|  | 11/06 – Search 3 | (((Corneal Transplantation) OR (Keratoplasty)) AND (Methods)) AND (Eye Banks) | 695 |
|  | 11/06 – Search 6 | ((((Corneal Transplantation) OR (Keratoplasty)) AND (Methods)) AND (Eye Bank)) AND (Cost) | 34 |
|  | 11/06 –  Search 7 | ((((Corneal Transplantation) OR (Keratoplasty)) AND (Procedure)) AND (Eye Bank)) AND (Cost) | 35 |
|  | 11/06 –  Search 9 | (((Corneal Transplantation) OR (Keratoplasty)) AND (Processing)) AND (Eye Bank) | 200 |
|  | 11/06 –  Search 10 | ((((Corneal Transplantation) OR (Keratoplasty)) AND (Processing)) AND (Eye Bank)) AND (Cost) | 7 |
| Total | 1.688 | | |
|  | | | |
| Embase | 11/06 –  Search 1 | ('cornea transplantation'/exp OR 'cornea transplantation' OR 'keratoplasty'/exp OR 'keratoplasty') AND ('procedures'/exp OR 'procedures') AND ('cost'/exp OR 'cost') AND ('eye bank'/exp OR 'eye bank') | 42 |
|  | 17/06  Search 2 | ('cornea transplantation'/exp OR 'cornea transplantation' OR 'keratoplasty') AND 'procedures' AND 'eye bank' | 246 |
|  | 17/06 –  Search 3 | ('cornea transplantation'/exp OR 'cornea transplantation' OR 'keratoplasty') AND method AND 'eye bank' | 200 |
|  | 11/06 –  Search 4 | ('cornea transplantation'/exp OR 'cornea transplantation' OR 'keratoplasty'/exp OR 'keratoplasty') AND ('methods'/exp OR methods) AND ('eye bank'/exp OR 'eye bank') AND ('cost'/exp OR 'cost') | 42 |
|  | 11/06 –  Search 5 | ('cornea transplantation'/exp OR 'cornea transplantation' OR 'keratoplasty'/exp OR 'keratoplasty') AND ('processing'/exp OR 'processing') AND ('eye bank'/exp OR 'eye bank') AND ('cost'/exp OR 'cost') | 3 |
|  | 11/06 –  Search 6 | ('cornea transplantation'/exp OR 'cornea transplantation' OR 'keratoplasty'/exp OR 'keratoplasty') AND ('processing'/exp OR 'processing') AND ('eye bank'/exp OR 'eye bank') | 108 |
| Total | 641 | | |
|  | | | |
| Scopus | 11/06 –  Search 1 | ALL ( ( "Corneal Transplantation" ) OR ( "Keratoplasty" ) ) AND ( "Procedure" ) AND ( "Costs" ) OR ( "Cost" ) AND ( "Eye Banks" ) OR ( "Eye Bank" ) | 188 |
|  | 11/06 –  Search 2 | ( ALL ( "Corneal Transplantation" ) OR TITLE-ABS-KEY ( "Keratoplasty" ) AND TITLE-ABS-KEY ( "Procedure" ) AND TITLE-ABS-KEY ( "Eye Banks" ) OR TITLE-ABS-KEY ( "Eye Bank" ) ) | 511 |
|  | 11/06 –  Search 4 | ( ALL ( "Corneal Transplantation" ) OR TITLE-ABS-KEY ( "Keratoplasty" ) AND TITLE-ABS-KEY ( "Methods" ) AND TITLE-ABS-KEY ( "Eye Banks" ) OR TITLE-ABS-KEY ( "Eye Bank" ) ) | 907 |
|  | 11/06 –  Search 6 | ALL ( ( "Corneal Transplantation" ) OR ( "Keratoplasty" ) ) AND ( "Methods" ) AND ( "Costs" ) OR ( "Cost" ) AND ( "Eye Banks" ) OR ( "Eye Bank" ) | 245 |
|  | 11/06 –  Search 7 | ALL ( ( "Corneal Transplantation" ) OR ( "Keratoplasty" ) ) AND ( "Processing" ) AND ( "Costs" ) OR ( "Cost" ) AND ( "Eye Banks" ) OR ( "Eye Bank" ) | 42 |
|  | 11/06 –  Search 9 | ( ALL ( "Corneal Transplantation" ) OR TITLE-ABS-KEY ( "Keratoplasty" ) AND TITLE-ABS-KEY ( "Processing" ) AND TITLE-ABS-KEY ( "Eye Banks" ) OR TITLE-ABS-KEY ( "Eye Bank" ) ) | 104 |
| Total | 1.997 | | |
|  | | | |
| Cochrane Library | 12/06 –  Search 1 | ("corneal transplantation") OR ("keratoplasty"):ti,ab,kw AND ("processing"):ti,ab,kw AND ("eye bank"):ti,ab,kw OR ("eye banks"):ti,ab,kw | 432 |
|  | 12/06 –  Search 2 | ("corneal transplantation") OR ("keratoplasty"):ti,ab,kw AND ("procedure"):ti,ab,kw AND ("eye bank"):ti,ab,kw OR ("eye banks"):ti,ab,kw | 432 |
|  | 12/06 –  Search 3 | ("corneal transplantation") OR ("keratoplasty"):ti,ab,kw AND ("methods"):ti,ab,kw AND ("eye bank"):ti,ab,kw OR ("eye banks"):ti,ab,kw | 432 |
|  | 12/06 –  Search 4 | ("corneal transplantation") OR ("keratoplasty"):ti,ab,kw AND ("methods"):ti,ab,kw AND ("cost"):ti,ab,kw AND ("eye bank"):ti,ab,kw | 370 |
|  | 12/06 –  Search 5 | ("corneal transplantation") OR ("keratoplasty"):ti,ab,kw AND ("procedure"):ti,ab,kw AND ("cost"):ti,ab,kw AND ("eye bank"):ti,ab,kw | 370 |
|  | 12/06 –  Search 5 | ("corneal transplantation") OR ("keratoplasty"):ti,ab,kw AND ("Processing"):ti,ab,kw AND ("cost"):ti,ab,kw AND ("eye bank"):ti,ab,kw | 370 |
| Total | 2.406 | | |
|  | | | |
| Web of Science | 12/06 –  Search 1 | ((((((ALL=("Corneal Transplantation")) OR ALL=("Keratoplasty")) AND ALL=("Processing")) AND ALL=("Cost")) OR ALL=("Costs")) AND ALL=("Eye Bank")) OR ALL=("Eye Banks") | 569 |
|  | 12/06 –  Search 2 | ((((((ALL=("Corneal Transplantation")) OR ALL=("Keratoplasty")) AND ALL=("Procedure")) AND ALL=("Cost")) OR ALL=("Costs")) AND ALL=("Eye Bank")) OR ALL=("Eye Banks") | 571 |
|  | 12/06 –  Search 3 | ((((((ALL=("Corneal Transplantation")) OR ALL=("Keratoplasty")) AND ALL=("Methods")) AND ALL=("Cost")) OR ALL=("Costs")) AND ALL=("Eye Bank")) OR ALL=("Eye Banks") | 578 |
| Total | 1.718 | | |
|  | | | |
| EBSCOhost | 16/06 –  Search 1 | (("corneal transplantation" OR "keratoplasty") AND ("procedure") AND ("eye bank")) | 147 |
|  | 16/06 –  Search 2 | (("corneal transplantation" OR "keratoplasty") AND ("methods") AND ("eye bank")) | 671 |
|  | 16/06 –  Search 3 | (("corneal transplantation" OR "keratoplasty") AND ("processing") AND ("eye bank")) | 85 |
| Total | 903 | | |
|  | | | |
| Science Direct | 16/06 –  Search 1 | (("corneal transplantation" OR "keratoplasty") AND ("procedure") AND ("cost) AND ("eye bank")) | 373 |
|  | 16/06 –  Search 2 | (("corneal transplantation" OR "keratoplasty") AND ("methods") AND ("cost) AND ("eye bank")) | 375 |
|  | 16/06 –  Search 3 | (("corneal transplantation" OR "keratoplasty") AND ("processing") AND ("cost) AND ("eye bank")) | 289 |
| TOTAL | 1.037 | | |
|  | | | |
| BVS | 13/08 | ("Corneal Transplantation" OR "Keratoplasty" OR "Transplante de Córnea" OR "Trasplante de Córnea") AND ("Methods" OR "Procedure" OR "Processing" OR "Métodos" OR "Métodos") AND ("Cost" OR "Custos" OR "Costos") AND ("Eye Banks" OR "Bancos de Olhos" OR "Bancos de Ojos") | 2 |
| TOTAL | 2 | | |
|  | | | |
| TOTAL OF THE DATABASES | 10.392 | | |

**Table 5.** Search term combinations and number of records retrieved from other methods, Campo Grande/MS, Brazil, 2025.

| **IDENTIFICATION OF STUDIES VIA OTHER METHODS** | | | |
| --- | --- | --- | --- |
| **Google Scholar** | 16/06 –  Search 1 | (("Corneal Transplantation" OR "Keratoplasty") AND ("Procedure") AND ("Cost" OR "Costs") AND ("Eye Bank" OR "Eye Banks"))  * After conducting research and selecting the first 100 documents, it was observed that these were already present in other periodicals, so it was decided not to use them. | 1.240 (Only the first 100 were selected.) |
| **TOTAL** | 100 | | |
| **Open Access Theses and Dissertations - NDLTD** | 16/06 –  Search 1 | "Corneal Transplantation" OR "Keratoplasty" AND "Procedure" AND "Eye Bank" | 2 |
|  | 16/06 –  Search 2 | Corneal Transplantation OR Keratoplasty AND Procedure AND Eye Bank | 11 (However, only 6 were different from the previous survey.) |
|  | 16/06 –  Search 3 | "Corneal Transplantation" OR "Keratoplasty" AND "Methods" AND "Eye Bank" | 2 |
|  | 16/06 –  Search 4 | "Corneal Transplantation" OR "Keratoplasty" AND "Processing" AND "Eye Bank" | 1 |
| **TOTAL** | 11 | | |
|  | | | |
| **TOTAL** | 111 | | |
